# *Talaromyces columbinus* sp. nov., and Genealogical Concordance Analysis in *Talaromyces* Clade 2a

**DOI:** 10.1371/journal.pone.0078084

**Published:** 2013-10-30

**Authors:** Stephen W. Peterson, Željko Jurjević

**Affiliations:** 1 Bacterial Foodborne Pathogens and Mycology Research Unit, United States Department of Agriculture, Peoria, Illinois, United States of America; 2 EMSL Analytical, Inc., Cinnaminson, New Jersey, United States of America; University of Missouri, United States of America

## Abstract

During the course of mold surveys, a set of *Talaromyces* isolates were obtained that did not fit any described species. Phenotypic examination of these isolates showed that they were similar to *T. piceus* but differed in some growth characteristics. Multilocus DNA sequence data were obtained for the new isolates and some related species in the broader, more inclusive clade, and the data were analyzed using genealogical concordance. The new isolates are described as *Talaromyces columbinus.* From analysis of the related species, *Penicillium rugulosum* var. *atricolum* is given species status in *Talaromyces* as *T. atricola*. *Penicillium tardum* and *P. chrysitis* were showed to be synonyms of *T. rugulosus*. *Penicillium scorteum* and *T. phialosporus* were showed to be conspecific and under the rule of priority *T. scorteus* is the proper name for isolates previously known as *T. phialosporus*. *Talaromyces wortmanii* was showed to be distinct from *Penicillium concavorugulosum* and *T. variabilis* but the relationship of the latter two species remains unresolved. Examination of ITS sequences from GenBank showed that *T. columbinus* has previously been reported from human lung infections under the name *Penicillium piceum*.

## Introduction

The genus *Penicillium* is widely known and studied because of its impact on human activities [1]. Historically, the broad sense of *Penicillium* has contained two very different groups, the first group containing monoverticillate, furcate and terverticillate species with broad ampuliform conidiogenous cells, and the second group containing biverticillate species with acerose conidiogenous cells. Where known, the two groups produced two different types of teleomorphs, either closed structures characterized by walls containing pseudoparenchymatous cells (*Eupenicillium*) or ascocarps having loosely woven hyphae forming the wall (*Talaromyces*). Several investigators e.g., [2] demonstrated the phylogenetic distinction between these two groups of *Penicillium*. Under the Melbourne nomenclatural code [3] dual naming was revoked in favor of using a single name for a single species. Samson et al. [4] formally recognized the phylogenetic distinction of the species with acerose conidiogenous cells and/or loosely woven ascocarp walls by placing them in the genus *Talaromyces*. *Talaromyces* is based on phylogenetic relationships, not whether the teleomorphic stage is known. Among the species placed in *Talaromyces* was *Penicillium piceum* under the new combination *Talaromyces piceus*.

Pitt [1] regarded *Penicillium piceum* as a relatively uncommon but widely distributed species. The species has been reported as causal organism in certain human lung infections [5], [6] and has been examined as a potential source of extracellular catalase [7] and isochaetochromin [8]. *P. piceum* has also been investigated as an indicator species for use in forensic science [9]. New *Talaromyces* species are being discovered as the molecular tools for phylogenetics are more widely applied to this genus [10]–[12].

During the course of fungal surveys conducted by one of us (ZJ) certain isolates resembling *T. piceus* were found that did not perfectly fit the description [1] and were retained for further investigation. Subsequent detailed examination showed that these isolates were representative of a previously unknown species that we describe here as *Talaromyces columbinus*. *Talaromyces piceus* fits into a small clade within *Talaromyces* designated clade 2a by Samson et al. [4]. In assessing the positions of the species within this clade, a number of phenotype-based taxonomic hypotheses [1], [13]–[15] were also tested using genealogical concordance analysis [16].

## Materials and Methods

### Isolations

Isolation of strains from air was performed by using a single stage bio-aerosol impaction sampler (EMSL VP-400 Microbial Sampler) for the viable sampling of fungi. The sampler contains over 400 precision drilled holes and a base section for placement of the agar media. Air was passed through each sampler with a flow rate of 28.3 L/minute for 3 minutes. The media used for fungal isolations was malt extract agar.

### Culture Methods

Isolates (Table 1) were grown on Czapek yeast extract agar (CYA), CYA with 20% sucrose (CY20S), malt extract agar (MEA), oatmeal agar (OA), dichloran-glycerol agar (DG18), Czapek yeast autolysate agar with 5% NaCl (CYA–5% NaCl), and creatine agar (CREA) [1], [17]. Cultures were incubated in darkness at 25°C for 7 d. Additional CYA cultures were incubated at different temperatures to determine the cardinal growth temperatures of the new species (10, 14.5, 20, 25, 30, 35, 37, 41, 42.5, 44.5, 46, 48 and 50°C) for 7 d. The cultures were grown on one plate as a three-point inoculation on each medium in 9 cm diam Petri dishes. Colony diameters and appearances were recorded and photographs were made from 7 d culture plates. Analysis of the growth data was performed using Sygraph in SYSTAT 11 for Windows [18]. The Ridgway [19] color guide was used to specify certain colors and those are referred to by plate number, e.g. (R47). Weighted regression analysis was used to model and compare the growth of 2 fungal species, *Talaromyces piceus* and *T. columbinus*, using the mean of 6 replications of colony diameter measurements (mm) as a function of temperature (°C). Standard weighting of 1/variance was used for each mean colony diameter at each temperature tested. A full and reduced model F-test was used to determine if the equations for each species were significantly different from one another. If a significant F-test value was obtained (indicating *T. piceus* was different from *T. columbinus* colony growth), regression coefficients were compared between the 2 species as well as colony diameters at each temperature using 95% confidence interval overlap.

**Table 1:** Provenance, GenBank accession numbers for DNA sequences and MycoBank numbers for species.

NRRL	Provenance	*BT*	*CF*	ITS	*Mcm7*	*RPB2*	*RPB1*	*Tsr1*
*Talaromyces allahabadensis* (B.S.Mehrotra & D. Kumar) Samson, Yilmaz & Frisvad MB 560640								
3397	India, isol ex soil. Ex type.	KF196856	KF196873	KF196910	KF196942	KF196973	KF196951	–
*Talaromyces atricola* S. W. Peterson and Ž. Jurjević MB 804733								
1052	From Bainier to Thom. Type isolate of *P. rugulosum* var. *atricolum*. Ex type of *T. atricola*.	KF196849	KF196872	KF196905	KF196943	KF196967	KF196958	KF196995
*Talaromyces columbinus* S. W. Peterson and Ž. Jurjević MB 804734								
35855	USA, California, isol ex air, *Z. Jurjevic*, January 2007.	KF196862	KF196883	KF196900	KF196926	KF196983	KF196948	–
35856	USA, California, isol ex air, *Z. Jurjevic*, January 2007.	KF196863	KF196884	KF196896	KF196927	KF196984	–	KF196996
35864	USA, California, isol ex air, *Z. Jurjevic*, May 2007.	KF196864	KF196885	KF196897	KF196928	KF196985	–	KF196998
58464	USA, New Jersey, isol ex air, *Z. Jurjevic*, July 2008.	KF196841	KF196881	KF196898	KF196929	KF196986	–	KF196999
58644	USA, Maryland, isol ex air, *Z. Jurjevic*, February 2009.	KF196842	KF196880	KF196899	KF196930	KF196987	–	–
58811	USA, Louisiana, isol ex air, *Z. Jurjevic*, October 2008, ex holotype.	KF196843	–	–	KF196931	–	–	KF196997
62680	USA, Illinois, isol ex corn grits (*Zea mays*), *D. I. Fennell*, June 1973.	KF196844	KF196882	KF196901	–	KF196988	KF196949	–
*Penicillium concavorugulosum* S. Abe MB 159173								
6192	Japan, isol ex soil. Abe’s type isolate.	KF196854	KF196867	KF196916	KF196939	KF196976	KF196959	KF196004
*Talaromyces islandicus* (Sopp) Samson, Yilmaz, Frisvad & Seifert MB 560654								
1036	South Africa, Capetown. Substrate unknown. Ex type.	–	KF196876	KF196918	KF196945	KF196969	KF196952	KF196000
*Talaromyces loliensis* (Pitt) Samson, Yilmaz & Frisvad MB 560655								
13064	New Zealand, isol ex rye grass. Ex type.	–	KF196877	KF196911	KF196946	KF196970	–	KF196001
*Talaromyces piceus* (Raper & Fennell) Samson, Yilmaz, Frisvad & Seifert MB 560661								
1051	USA, isol by *C. W. Emmons*, to Thom 5623.6, to NRRL, 1937; ex neotype.	KF196845	KF196886	KF196893	KF196920	KF196978	–	–
1071	subculture of NRRL 1051	KF196846	KF196887	–	KF196921	KF196979	–	–
2112	Canada, Saskatchewan, isol ex alfalfa seed, *J. W. Groves*, 1945.	KF196847	KF196888	KF196894	KF196922	KF196980	–	–
13017	USA, Texas, isol ex scarab dung ball, *D. T. Wicklow*, February 1982.	KF196848	–	–	KF196924	–	–	–
35764	USA, California, isol ex air, *Z. Jurjevic*, March 2007.	KF196865	KF196889	KF196892	KF196923	KF196981	–	–
35955	USA, Louisiana, isol ex air, *Z. Jurjevic*, February 2008.	KF196866	KF196890	KF196895	KF196925	KF196982	–	–
*Talaromyces proteolyticus* (Kamyschko) Samson, Yilmaz & Frisvad MB 560665								
3378	Russia, Leningrad, isol ex soil. Ex type.	KF196857	–	KF196919	KF196947	KF196989	KF196960	KF196005
*Talaromyces radicus* (A. D. Hocking & Whitelaw) Samson, Yilmaz, Frisvad & Seifert MB 560669								
1069	Derived from Thom 5403.1.	KF196855	KF196870	KF196913	KF196941	KF196971	KF196950	KF196990
29340	Australia, rhizosphere of wheat. Ex type isolate.	–	KF196871	KF196912	–	KF196972	–	KF196991
*Talaromyces rotundus* (Raper & Fennell) C. R. Benj. MB 306719								
2107	Panama, Chiriquí, isol ex wood, *G. W. Martin*. Ex type.	KF196861	–	KF196906	–	KF196968	–	–
*Talaromyces rugulosus* (Thom) Samson, Yilmaz, Frisvad & Seifert MB 560672								
1045	USA, Connecticut, isol ex potato tuber, *C. Thom*. Ex type.	KF196858	KF196868	KF196902	KF196937	KF196964	KF196955	KF196992
1053	Unknown. Biourge type isolate of *Penicillium chrysitis*.	KF196859	KF196869	KF196904	KF196935	KF196965	KF196957	KF196994
1073	France, isol ex twigs, type isolate of *Penicillium tardum*.	KF196860	–	KF196903	KF196936	KF196966	KF196956	KF196993
*Talaromyces scorteus* (Nakazawa, Takeda, & Suematsu) S. W. Peterson and Ž. Jurjević MB 804734								
203	USA, California, isol ex milled rice. Type isolate of *P. phialosporum*.	KF196850	KF196874	KF196907	KF196932	KF196962	–	KF196003
1129	Japan, isol military equipment. Ex neotype isolate of *T. scorteus*.	KF196851	–	KF196908	KF196933	KF196961	KF196953	–
2117	Unknown, isol by *W. H. Weston*, ca 1945.	KF196852	KF196875	KF196909	KF196934	KF196963	KF196954	–
*Talaromyces variabilis* (Sopp) Samson, Yilmaz, Frisvad & Seifert MB 560676								
1048	South Africa, Johannesburg, isol ex coconut matting, *J.W. Bowen*. Ex type.	KF196853	KF196878	KF196915	KF196938	KF196975	–	–
*Talaromyces wortmanii* (Klöcker) C. R. Benj. MB 344294								
1017	Denmark, isol ex soil. Ex type.	–	KF196879	KF196914	KF196940	KF196974	–	–
*Talaromyces* sp.								
62223	USA, North Carolina, isol ex corn, *R. Rogers*.	–	KF196861	KF196917	KF196944	KF196977	–	KF197002

### Microscopy

Microscopic examination was performed by gently pressing a ca 20×5 mm piece of transparent tape onto a colony, rinsing the tape with one or two drops of 70% ethanol and mounting the tape in lactic acid with fuchsin dye. Additional microscopic samples were made by teasing apart a small amount of mycelium in a drop of water containing 0.5% Tween 20. A Leica DM 2500 microscope with bright field, phase contrast and DIC optics was used to view the slides. A Spot camera with Spot imaging software was mounted on the microscope and used for photomicrography. A Nikon digital SLR camera with a D70 lens was used for colony photography. Photographs were re-sized and fitted into plates using Microsoft PowerPoint 2010.

### Phylogenetic Analysis

Strains used in phylogenetic analysis (Table 1) were grown in 25 mL of 2% malt extract broth in 125-mL Erlenmeyer flasks shaken at 200 rpm (25–28°C). Mycelium was harvested after 1–2 days growth by vacuum filtration over Whatman #1 filter paper and then placed loosely in microfuge tubes, frozen and freeze dried. The freeze-dried biomass was ground to a fine powder and rehydrated with 0.5 mL CTAB buffer [20]. Proteins were extracted by the addition of 0.5 mL chloroform. After brief emulsification, the aqueous phase was separated from the organic phase by centrifugation. The aqueous phase was transferred to a clean tube and DNA was precipitated by the addition of 0.5 mL isopropanol. The precipitate was collected by centrifugation and rinsed with 70% ethanol. The pellet was rehydrated with 0.1 mL TE buffer and stored at −20°C until used.

Beta tubulin (*BT2*), calmodulin (*CF*), nuclear internal transcribed spacer region (ITS), DNA replication licensing protein (*Mcm7*), RNA polymerase II (*RPB2*) and ribosome biogenesis protein (*Tsr1*) were amplified from 1∶10 diluted genomic DNA using previously described primers and conditions [21]. RNA polymerase II largest subunit (*RPB1*) sequences were obtained for select isolates using published methods [4]. Initial results indicated that the *BT2* primers (BT2a, BT2b) were amplifying paralogous genes. The primers (BT2f and T22) and procedures of Hubka and Kolarik [22], developed to resolve this problem in *Aspergillus japonicus,* were used to conduct repeated amplification and sequencing of the suspect products. This procedure also produced the paralogous gene products.

Amplified DNA was prepared for sequencing reactions with ExoSapit [23]. Sequencing reactions were performed using DyeDeoxy terminator kits (v 3) following the manufacturer’s instructions and subsequent analysis on an ABI 3730 DNA analyzer [24]. Sequencing was performed in both directions and any conflicts were resolved using Sequencher 5 [25] to visualize the sequences. Sequences were carefully reviewed for quality and experiments were repeated if there was doubt about the reliability of sequence reads. Sequences were subjected to Blast search against the GenBank nr database.

Three DNA sequence datasets were aligned using Clustalw [26]. One was composed of a wide array of species in the genus *Talaromyces* clade 2A [4] including only *RPB2* data, whose purpose was to show the overall position of the new species in the clade. The other alignments were sequences from *T. piceus* and *T. columbinus* isolates for each of the sequenced genes and alignments of *T. rugulosus* and *T. phialosporus* and putative synonyms. Aligned datasets were analyzed using maximum parsimony [27] and 1000 bootstraps. Conditions for parsimony analysis were random sequence addition, maxtrees = 5000 and swap = tbr (tree bisection and reconnection). For bootstrap calculations addseq = asis, maxtrees = 100 and swap = tbr were set. *Talaromyces proteolyticus* was used as the out-group species for the analysis based on the study by Samson et al. [4]. Tree files were visualized and converted to emf format using TreeView [28] and redrawn for publication using CorelDraw X6 [29]. Genealogical concordance [16] was assessed through visual comparison of the individual locus trees.

## Results

### DNA Sequences

DNA sequences determined in this study are deposited in GenBank and accession numbers are found in Table 1.

Blast searches of GenBank using ITS sequences revealed that *T. piceus* ex type isolates held by NRRL (Agricultural Research Service culture collection Peoria, IL), CBS (Centraalbureau voor Schimmelcultures, Utrecht, The Netherlands) and ATCC (American Type Culture Collection, Manassas, VA) that originated from the same parental culture were different. CBS 361.48 (GenBank JN899370 ) differs from NRRL 1051 (GenBank KF196893) by six gap positions, all deletions of T residues relative to NRRL 1051; ATCC 10519 (GenBank DQ666826) differs from NRRL 1051 by insertion of two A residues and one T residue relative to NRRL 1051. Five isolates of *T. piceus* examined in this study (Table 1) shared an identical and unique ITS sequence. The *RPB2* sequence of CBS 391.48 (JF417433) should be identical to NRRL 1048 (KF196975) because the cultures originated from the same parental culture. Of 975 comparable bases, there is an A/G difference near the 3′ end of the read. The RPB1 sequence for *P. tardum* NRRL 1073 generated here (KF196957) differs at 94 out of 455 nucleotide positions from the sequence of CBS 258.37 (JN899293). These isolates are putatively descended from the same parent culture.

Blast searches of our new species against the GenBank nr (non-redundant sequence) database provided a 100% similarity of our new species to the ITS sequence from IMI 392509 (DQ666824) (IMI, International Mycological Institute, Egham, UK), a fungus isolated from a human lung infection in Buenos Aires, Argentina [6]. Other gene sequences from our new species (*BT2*, *CF, Mcm7, RPB2, Tsr1* and *RPB1*) produced no high homology to any sequences in the nr database.

Seven of eight *T. columbinus* isolates shared an identical ITS sequence (e.g. KF196896) and one isolate had a single transition (KF196900) versus the others. *T. piceus* isolates shared the same ITS sequence (e.g., KF196893). The common species sequences differed by seven transition base differences, one transversion and one indel. In our analysis, barcode identification [30] of these species is practical.

**Figure 1:**
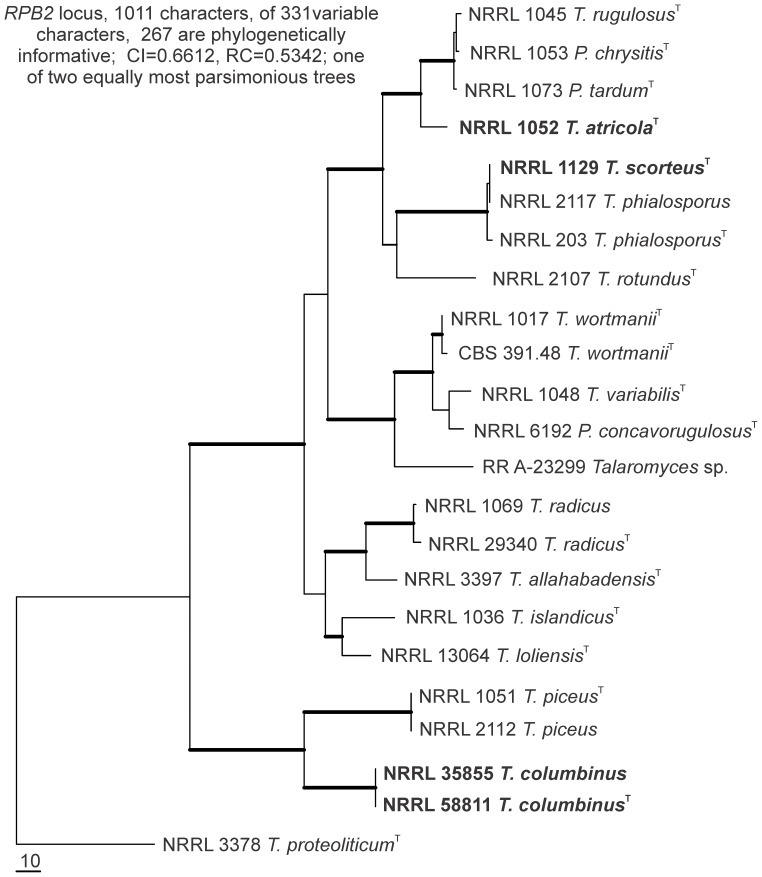
Phylogeny of *Talaromyces* clade 2a. Phylogenetic tree of species from part of *Talaromyces* based on maximum parsimony analysis of *RPB2* gene sequences. Bootstrap values above 90% are shown as thick lines in the tree. *T. columbinus* and *T. piceus* form strongly supported branches; *T. rugulosus, P. chrysitis* and *P. tardum* form a strongly supported branch with *T. atricola* as sibling; *T. scorteus* and *T. phialosporus* form a strongly supported branch. Species on a strongly supported branch that are not well distinguished are potentially synonymous. The tree is rooted with *T. proteolyticus* on the basis of prior more comprehensive analysis of the genus.

**Figure 2:**
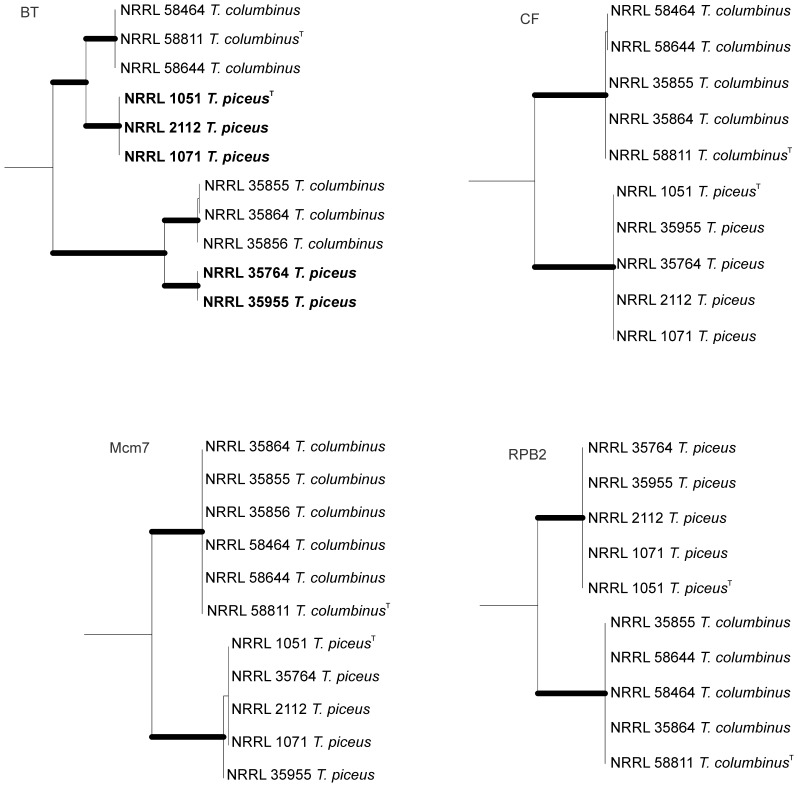
Genealogical analysis of *T. piceus* and *T. columbinus*. Excised portions of phylogenetic trees based on beta tubulin (*BT2*), calmodulin (*CF*), minichromosome maintenance factor 7 (*Mcm7*) and RNA polymerase beta (*RPB2*). Bootstrap values above 90% are represented as bold lines. The *BT2* tree divides *T. piceus* and *T. columbinus* isolates into four statistically supported clades while the other three loci place all *T. piceus* isolates on a single branch and all *T. columbinus* isolates on another branch. The *BT2* primer set appears to be amplifying different gene fragments or the locus may have undergone rearrangements that make the analysis appear paralogous. Genealogical concordance is seen in a majority of the trees, which supports *T. columbinus* and *T. piceus* as distinct species.

The *RPB2* based phylogeny of *Talaromyces* clade 2a is shown in Fig. 1. Phylogenetic relationships and topology of single locus trees of the isolates are shown in Fig. 2. The product of *BT2* primer amplifications placed isolates of *T. piceus* and *T. columbinus* in four statistically supported distinct clades. That tree is notably discordant from the *CF*, *Mcm7* and *RPB2* loci (Fig. 2) that place the same isolates in two strongly supported clades. The majority rule of congruence analysis indicates these are distinct species [16].

**Figure 3:**
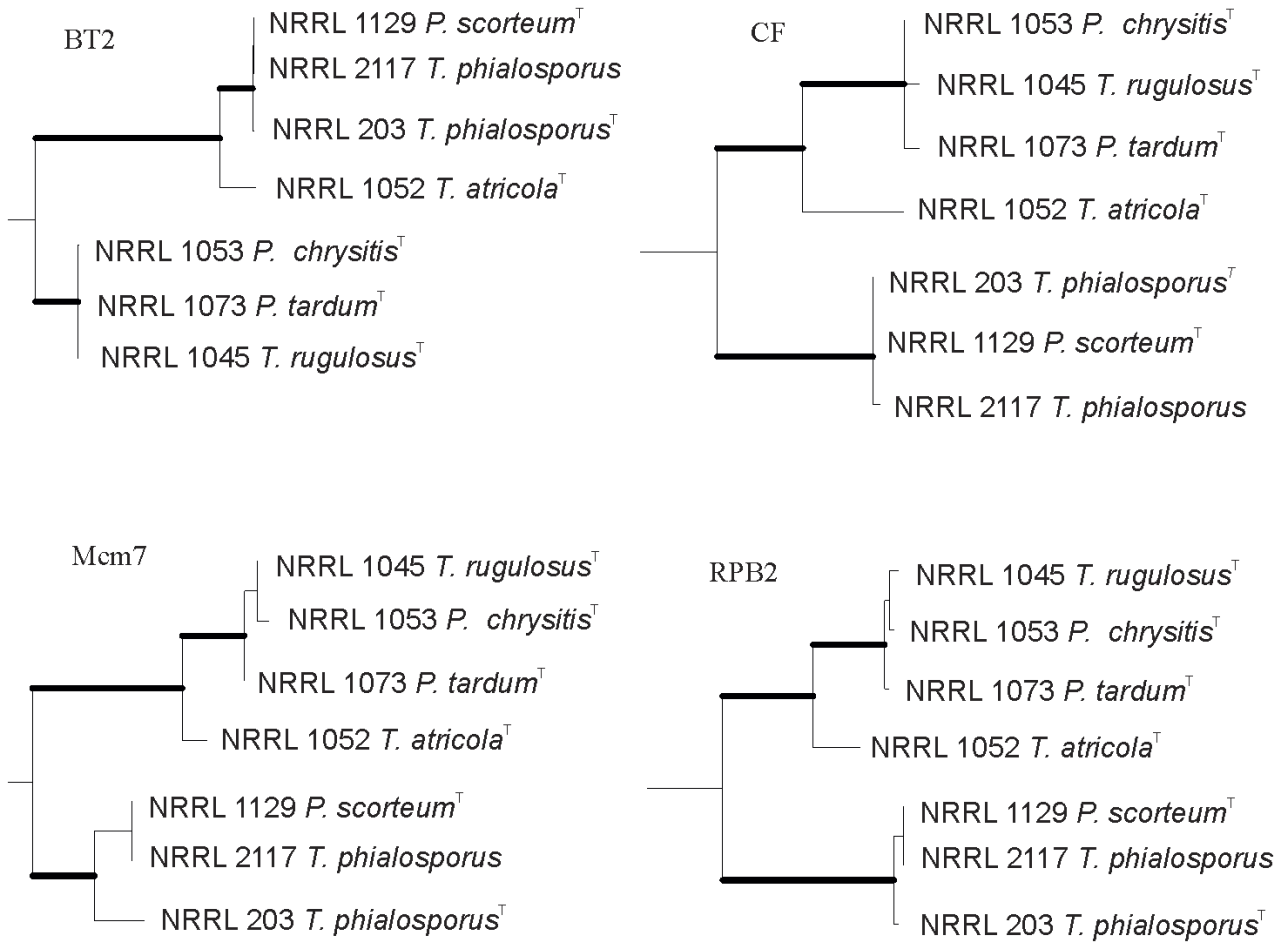
Genealogical concordance analysis. Excised portions of phylogenetic trees based on beta tubulin (*BT2*), calmodulin (*CF*), minichromosome maintenance factor 7 (*Mcm7*) and RNA polymerase beta (*RPB2*). Bootstrap values above 90% are represented as bold lines. *Talaromyces atricola* is sibling to *T. rugulosus* at each locus supporting it as a distinct species. *Penicillium scorteum* has in the past been considered a synonym of *T. rugulosus* but this analysis shows that *P. scorteum* is conspecific with *T. phialosporus.*

The phylogenetic relationships of *T. phialosporus* and *Penicillium scorteum,* and *T. atricola, T. rugulosus*, *P. chrysitis* and *P. tardum* are shown in Fig. 3. *T. phialosporus* and *P. scorteum* are conspecific; *T. rugulosus P. tardum* and *P. chrysitis* are conspecific, with strong support from concordance analysis and *T. atricola* is a distinct species.

**Figure 4:**
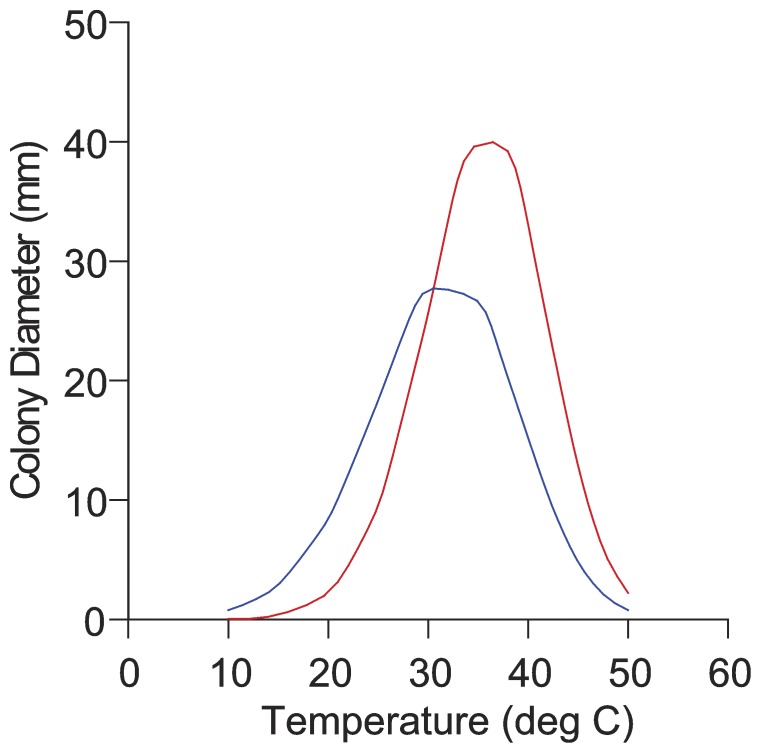
Temperature dependent growth. Graphical depiction of temperature dependent colony diameter for isolates of *T. piceus* (blue line) and *T. columbinus* (red line). *T. piceus* growth begins at 14.5°C, *T. columbinus* begins growth at 20°C, at the upper temperature range (44.5°C) *T. columbinus* colonies are ca 20 mm diam, while *T. piceus* isolates mostly do not grow. *T. columbinus* isolates tend to greater colony diameter at most temperatures but with great variation among the isolates.

*Temperature and medium dependent colony growth:*–Full and reduced model comparison analysis of temperature dependent growth rates (Fig. 4) showed that the equations for *T. piceus* and *T. columbinus* were significantly different from each other at α = .01 and.05 levels. Confidence interval overlap on the regression coefficients a, b, and c showed that intercepts (a) and slopes for X (b) and X^3^ (c) aren’t different between the equations for the 2 species. It appears that *T. columbinus* and *T. piceus* show the same colony growth at the lower and higher temperatures extremes tested (10 and 50°C) with growth lagging for *T. columbinus* at 14.5 through 30°C, then surpassing *T. piceus* at temperatures 35 through 48°C. *Talaromyces columbinus* isolates showed no growth below 20°C, while *T. piceus* isolates all produced small colonies at 14.5°C. Maximum growth appears at ca 35°C, where the growth of *T. columbinus* exceeds that of *T. piceus* although the individual isolates are variable. At 44.5°C *T. piceus* isolates showed little or no growth while *T. columbinus* isolates’ colonies were 10–20 mm diam.

Colony diameters of *T. piceus* and *T. columbinus* isolates grown for 7d at 25°C on the different media were: for *T. columbinus*|*T. piceus* on **MEA** 10–20 mm|21–27 mm; on **OA** 10–17 mm|16–22 mm; on **CY20S** 3–12 mm|10–17 mm; on **CYA–5% NaCl** no growth|3–15 mm; on **DG18** 5–7 mm|8–12 mm; and on **CREA** 5–8 mm|14–18 mm with no acid production in either species.

### Taxonomy

**Figure 5:**
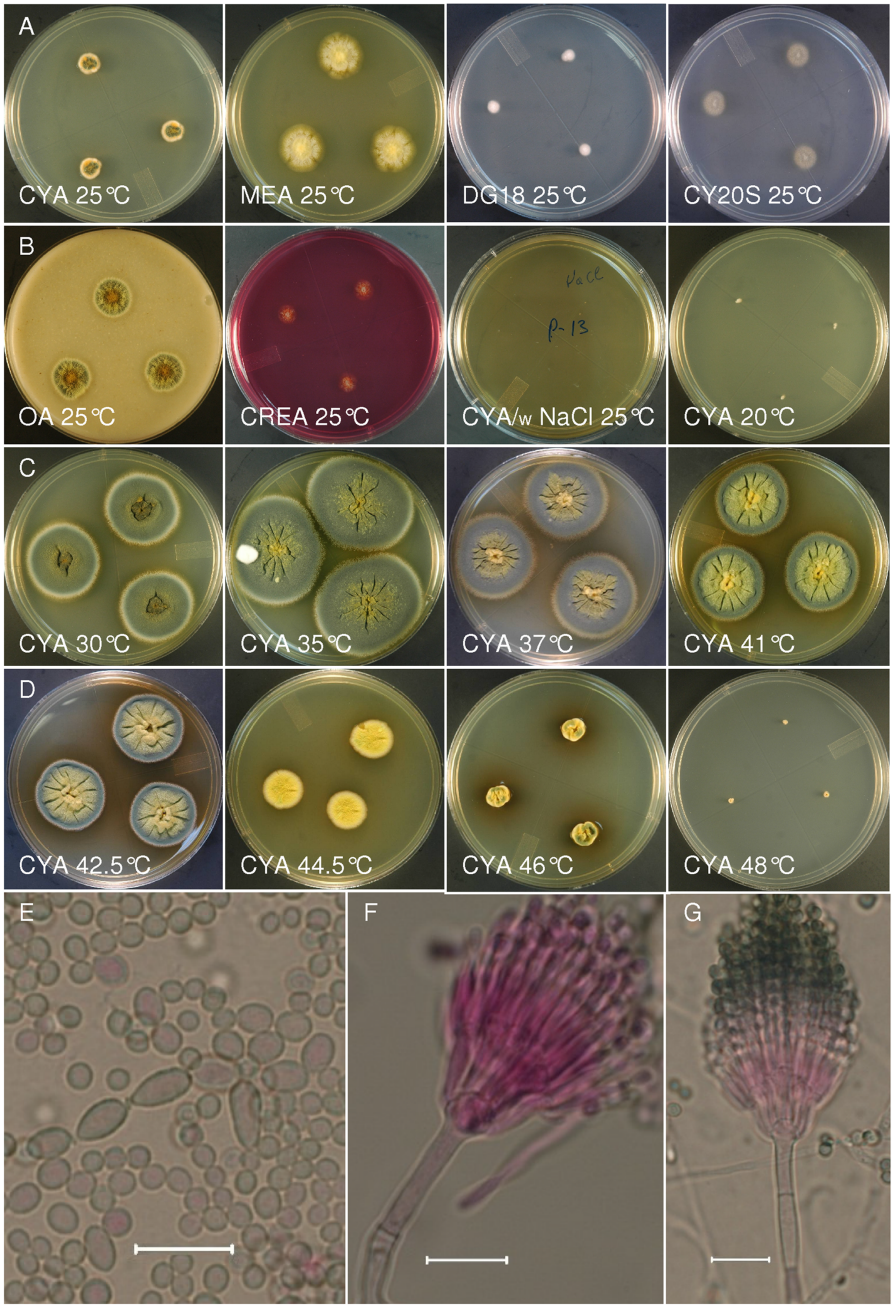
Talaromyces columbinus. Panels A–D show colonies of NRRL 58811 grown 7d with the specified medium and incubation temperature. There is no growth on NaCl amended CYA medium. Cultures incubated at temperature between 30°C and 46°C produce a dark brown soluble pigment in the agar. The color of the CYA culture at 37°C is the basis of the epithet. E. Conidia mostly globose 2.5–3.5 µm diam and smooth walled with occasional much larger and ovoid individuals, bar = 10 µm. F, G. Penicillus structure with vesiculate stipe, metulae and phialides. Conidia often form conical aggregations apically on the penicillus, bar = 10 µm.

***Talaromyces columbinus*** S.W. Peterson and Ž. Jurjević sp. nov. (Fig. 5).

[urn:lsid:indexfungorum.org:names: Mycobank: MB 804732.

***Etymology:*** the epithet refers to the dark bluish-gray colony color on certain media.

***Holotype:*** BPI 892668, a dried culture of NRRL 58811, USA, Louisiana, isol ex air sampler, Ž. *Jurjević*, October 2008.

***Diagnosis*** **:** Microscopically similar to *T. piceu*s, producing colonies on CYA with a distinct dark bluish gray color, and growing well at 44.5°C, versus minimal or absent growth at 44.5°C for *T. piceus*; *T. columbinus* isolates do not grow on CYA–5% NaCl while *T. piceus* isolates grow moderately well.

***Description*** **:** CYA, 7 d, 25°C, colony diam 6–15 mm, conidial area, pea green to Artemisia green (R47); good to abundant conidium production, mycelium overgrown with aerial hyphae in light orange-yellow to orange (R3) shades, periphery light yellow orange occasionally white; velutinous to lightly floccose, centrally rising ca 2–4 mm, occasionally radially sulcate, margin submerged ca 1 mm, exudate when produced clear, yellowish brown; soluble pigment when present faint brownish shades, no sclerotia; reverse xanthine orange to amber brown (R3); MEA, 7 d, 25°C, colony diam 10–27 mm diam, conidial area is Vetiver green to Artemisia green (R47), or bluish green to dark bluish green shades near deep Medici blue (R48), overgrown with low thin, scattered pigmented mycelial fascicles of straw yellow to wax yellow (R16) shades, conidium production abundant to very abundant, low and plane, centrally raised ca 3–4 mm in a cushion ca 3–4 mm diameter; peripherally subsurface or submerged hyphae ca 3–6 mm, lemon yellow (R4), exudate when produced after 10 d yellowish to brownish, sparse, soluble pigment not seen, no sclerotia; reverse xanthine orange (R3) to apricot yellow (R4) peripherally; CY20S, 7 d, 25°C, colony diam (3–)10–12 mm, conidial area pea green to Artemisia green (R47); conidium production abundant to very abundant, mycelium white to crème; reverse uncolored; OM, 7 d, 25°C, colony diam (10–)15–17 mm, conidial area green to olive-green to deep turtle green (R32), conidium production very abundant, mycelium light orange yellow to xanthine orange (R3) centrally, occasionally white to orange yellow at margins, exudate pale yellow occasionally clear to amber brown (R3), commonly abundant, occasionally centrally rising ca 3–5 mm; CREA, 7 d, 25°C, colony diam 5–8 mm, conidial area olive-green, conidium production good, no acid production; DG18, 7 d, 25°C, colony diam 5–6 mm, conidial area cream to light buff (R15), sporulation good, mycelium white, reverse light buff (R15); CYA–5% NaCl, 7 d, 25°C, no growth.

*Stipes* arise from surface or aerial hyphae, rarely from rope-like hyphal formations, (5–)15–35 (–75)×2.5–3.5(–4.5) µm, with smooth walls, terminally inflated (3–)4–6(–10) µm diam, bearing terminal biverticillate, occasionally monoverticillate or more complex penicilli, metulae in appressed verticils of (3–)8–12, with terminal swelling up to 5 µm diam, 7–10(–12)×(2–)2.5–3.5(–4) µm, phialides 4–7 per metula, acerose 7–10(–12)×(1.5–)1.8–2.2(–2.6) µm, conidia spherical to ellipsoidal (2.2–)2.5–3.5(–8)×2.5–3.5(–5) µm, with smooth to finely roughened walls, borne in short close conical or pyramidal chains.

***Talaromyces atricola*** S. W. Peterson and Ž. Jurjević, *comb. et stat. nov.*

[urn:lsid:indexfungorum.org:names: Mycobank: MB 804733.

**Basionym**: *Penicillium rugulosum* var. *atricolum* (Bainier) Thom. The Penicillia 1930:474 (Mycobank MB277103).

Thom [15] described a culture received from Bainier labeled *Penicillium atricolum* as *Penicillium rugulosum* var. *atricolum* Thom. That culture exists as NRRL 1052 and proved to represent a species distinct from *P. rugulosum* (Figs. 1, 3). Accordingly this isolates is given the new name *Talaromyces atricola*. **Holotype** is the lyophilized culture NRRL 1052 received by Charles Thom and accessioned into his collection as 4640.439 and later accessioned into the ARS culture collection as NRRL 1052.

***Talaromyces scorteus*** (Nakazawa, Takeda, & Suematsu) S. W. Peterson and Ž. Jurjević, *comb. nov.* [urn:lsid:indexfungorum.org:names: Mycobank: MB 804734.

Basionym: *Penicillium scorteum* Nakazawa, Takeda and Suematsu, Journal of Agricultural Chemical Society of Japan 10:103. 1934.

≡ *Penicillium scorteum* Nakazawa et al.; MB 492647.

 =  *Penicillium phialosporum* Udagawa; MB 302415.

*=  Talaromyces phialosporus* (Udagawa) Samson, Yilmaz & Frisvad; MB560660.

**Neotype**: BPI 892679 dried colony preparation of NRRL 1129, isolated from military equipment, designated here.

Nakazawa and associates, in accordance with then current rules did not designate a type, but distributed a type isolate (CBS 340.34) which was later obtained by Thom and accessioned in the Agricultural Research Service culture collection as NRRL 1129. This isolate, as dried colonies is designated as the neotype.

Raper and Thom [14] reported the authors of *P. scorteum* as Takedo, Suematsu and Nakazawa. This error has been perpetuated in the literature e.g., [1], [14], [31]. An interpretation of the original article, which was written mostly in Japanese, reveals that the authors are Nakazawa, Takeda and Suematsu, fide Prof. Junta Sugiyama.

*Talaromyces rugulosus* (Thom) Samson, Yilmaz, Frisvad & Seifert, MB560672.

≡*Penicillium rugulosum* Thom; MB 210907.

 = *Penicillium tardum* Thom; MB 279778.

 = *Penicillium chrysitis* Biourge; MB260588.

## Discussion

The sequence differences between the three type isolates of *T. piceus* could arise from sequence reading errors, mishandling of the cultures so that they are no longer identical or on rare occasion copies of the rDNA repeat units have different sequences [32]. The multiple gaps distinguishing the ITS sequences of NRRL 1051 (KF196893), CBS 342.48 (JN899370) and ATCC 10519 (DQ666826) are most easily explained as sequence reading errors. The single base difference between the *RPB2* sequences of two *T. rugulosus* type isolates (NRRL 1048, KF196975 and CBS 391.48, JF417433) appears to be a simple sequence reading error. The ca 20% sequence difference between the *RPB1* sequence from two type isolates of *P. tardum* (NRRL 1073, KF196957 and CBS 258.37, JN899293) is too great to be simple sequencing error and most likely resulted from mishandling of sequence data or cultures. Samson et al. [4] indicated some concern about their sequence from this isolate.

*Talaromyces columbinus* isolates were collected in air samplers in several widely separated states of the USA and from a sample of corn grits in Illinois. It is widely dispersed in the US. The 100% similarity of the ITS sequence from the lung infection fungus of a patient in Buenos Aires (IMI 392509, DQ666824) with *T. columbinus* (KF196900) suggests that this infective species is *T. columbinus* and that the species is present in Argentina, although we have not seen the actual isolate. The patient in Argentina [6] had underlying chronic granulomatous disease that appears to make patients susceptible to opportunistic fungal infection [33]. Another lung infection fungus from a patient in Bonn, Germany [5] was identified by ITS sequence and phenotype. The phenotype suggested *P. piceum* and the ITS sequence was reported to be similar to that of *P. piceum* CBS 361.48 but it was not deposited in a public database. The authors of that study [5] made the sequence available to us and it is a 100% match to the sequence from *T. columbinus*. On this basis we are confident that the German lung infection isolate was *T. columbinus*. On the basis of 100% ITS sequence similarity, *T. columbinus* is known from North America, South America and Western Europe.

**Figure 6:**
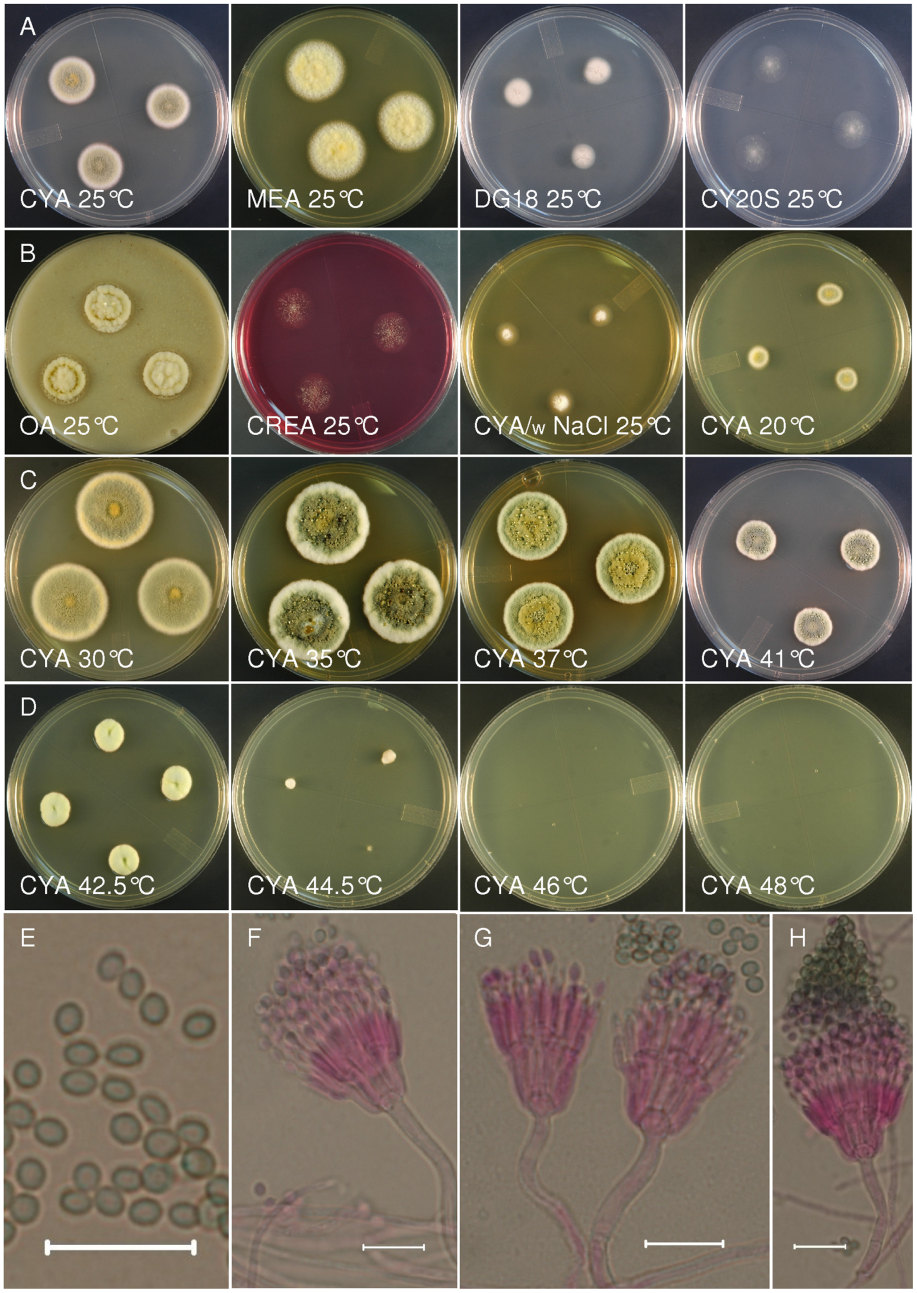
Talaromyces piceus. Panels A–D show colonies of NRRL 1051 grown 7 d with the specified medium and incubation temperature. Incubation at 37°C on CYA does not produce the bluish-gray color seen in *T. columbinus* and exudate is evident on colonies grown at 35–41°C. E. Conidia, subglobose to ellipsoidal, smooth walled 2–2.5×3–3.5 µm. F–H. Penicillus structure very similar to that of *T. columbinus* including the conical aggregation of conidia at the apex of the penicillus. Bar = 10 µm.

The phenotypic distinction of *T. piceus* and *T. columbinus* relies most notably on the failure of *T. columbinus* isolates to grow on CYA medium supplemented with 5% NaCl, and by the color of colonies grown at 37°C on CYA (compare Figs. 5, 6). The lack of growth on NaCl supplemented medium appears to be a reaction to the salt rather than an osmotic effect since *T. piceus* and *T. columbinus* showed moderate growth on DG18 medium (18% glycerol) and CY20S (20% sucrose). Both species grow above 37°C, but *T. columbinus* grows well at 44°C while *T. piceus* isolates mostly fail to grow at this temperature. Colony diameters in the middle area of the temperature growth range show differences by species (Fig. 4) but the variation seen in the isolates of each species makes reliance on this one character dubious.

Genealogical concordance analysis of multilocus DNA sequence data from isolates of *Talaromyces rugulosus*, *Penicillium chrysitis* and *P. tardum* revealed that they are conspecific. *Penicillium rugulosum*, the basionym of *T. rugulosus* was published in 1910 (MB 210907) and has priority over *P. chrysitis* Biourge published in 1923 (MB 260588) and *P. tardum* Thom published in 1930 (MB 279778). Samson et al. [4] placed *P. tardum* in their tree diagram in a different clade along with *T. pinophilus*. We carefully checked our data and fungal isolates and are confident that our placement of *P. tardum* in synonymy with *T. rugulosus* is correct.

*Penicillium rugulosum* var. *atricolum* Thom (MB 274357) was found in concordance analysis to represent a distinct species sibling to *T. rugulosus* and has been elevated to species status in *Talaromyces* as *T. atricola*.

Fungal nomenclature is based on type specimens and publication of names based on those types. If two people should describe the same new species under different names, the name proposed by the author first to publish is chosen as the legitimate name using the principle of priority. There are provisions in the nomenclatural code for conserving a name that may be synonymous with an earlier published name. Generally a name can be conserved if it is associated with significant industrial processes or very widely embedded in literature. A strong case for the advantages of keeping a later synonym as the legitimate name must be made and ruled on [34]. Frisvad et al. [35] proposed preserving the names *Penicillium chrysogenum* (penicillin producer), *Aspergillus niger* (enzyme and citrate producer) and *Aspergillus nidulans* (a model genetic system) and protected status was granted [34]. Slightly later Pitt and Samson [31] generated a comprehensive list of commonly used names in *Aspergillus* and *Penicillium* and asked for protected status for all those names. Protection for the names was not granted [34], but following the suggestions of Pitt and Samson [31] was recommended to promote taxonomic stability.

*Penicillium scorteum* is listed by Raper and Thom [14], Pitt [1] and Pitt et al. [13] as a synonym of *P. rugulosum*. However as our data analysis (Fig. 3) shows *P. scorteum* is conspecific with *T. phialosporus*. Publication of *P. scorteum* in 1934 (MB 492647) predates the publication of *P. phialosporum* in 1959 (MB 302415). *Talaromyces phialosporus* is accepted in the list of names in common use (NCU) [31]. However, *Talaromyces phialosporus* is not a commonly encountered species [36] and no widely known industrial or medical process is associated with this name. *Penicillium scorteum* is also a name not often encountered. In this case we adhere to the rule of priority rather than the NCU as neither *P. scorteum* nor *T. phialosporus* are commonly used or reported names and this name change will not have widespread disadvantageous effect.

Samson et al. [4] presented a tree diagram based on *RPB1* sequences that portrayed *T. variabilis, P. concavorugulosum* and *T. wortmanii* as identical. In Fig. 1 there is a clear distinction of each of these species based on *RPB2* sequences. Strong statistical support shows that *T. wortmanii* is a distinct species, but additional analysis based on more isolates and more loci is required to establish the relationship of *T. variabilis* and *P. concavorugulosum* under the genealogical concordance paradigm.

The electronic version of this article in Portable Document Format (PDF) in a work with an ISSN or ISBN will represent a published work according to the International Code of Nomenclature for algae, fungi, and plants, and hence the new names contained in the electronic publication of a PLOS ONE article are effectively published under that Code from the electronic edition alone, so there is no longer any need to provide printed copies.

In addition, new names contained in this work have been submitted to MycoBank from where they will be made available to the Global Names Index. The unique MycoBank number can be resolved and the associated information viewed through any standard web browser by appending the MycoBank number contained in this publication to the prefix http://www.mycobank.org/MycoTaxo.aspx?Link=T&Rec=. The online version of this work is archived and available from the following digital repositories: PubMed Central, LOCKSS.
